# Storage diseases with hypertrophic cardiomyopathy phenotype

**DOI:** 10.21542/gcsp.2018.28

**Published:** 2018-08-12

**Authors:** Luis Ruiz-Guerrero, Roberto Barriales-Villa

**Affiliations:** 1 Cardiology Service, Hospital Universitario Marqués de Valdecilla, Instituto de Investigación Marqués de Valdecilla (IDIVAL), Santander, Spain; 2 Inherited Cardiovascular Diseases Unit, Cardiology Service, Complexo Hospitalario Universitario de A Coruña, Instituto de Investigación Biomédica de A Coruña (INIBIC), Servizo Galego de Saúde (SERGAS), Universidade da Coruña, A Coruña, Spain; 3 Centro de Investigación Biomédica en Red. Enfermedades Cardiovasculares (CIBERCV), Madrid, Spain

## Abstract

Never judge a book by its cover, nor assume hypertrophic cardiomyopathy (HCM) as sarcomeric, as appearances can deceive. HCM phenocopies account for a 5–10% of the cases, mainly represented by storage diseases, flagged by the increasing prevalence of senile cardiac amyloid in developing countries. Multisystemic and heterogeneous presentation of these entities is a challenge for clinicians, and time delay in diagnosis is a major concern. Promising drugs and gene-specific tailored therapies are under development, therefore, more than ever, appropriate understanding of these conditions is mandatory for adequate early treatment and counselling.

In this review, storage disorders will be classified as extracellular and intracellular deposit storage diseases, focusing our attention on the most prevalent conditions from the cardiologist’s perspective.

## 1 Extracellular deposit storage

### 1.1 Cardiac amyloidosis (CA)

#### 1.1.1 Introduction and epidemiology

In the heart, amyloidosis can mimic hypertrophic cardiomyopathy and lead to systolic and diastolic dysfunction, as a result of the stiffness and poor compliance of the myocardium, the small ventricle cavity and the reduced diastolic contribution of infiltrated atriums. The hallmark of amyloidosis is the disruption of vital organ function through the deposition of extracellular fibrils, conformed by aggregate of proteins with unstable tertiary structures, misfolded and/or misassembled. Other particular aspects of this fibrils are that they are rigid, non-branching, linear, width of about 7.5 nm and indefinite in length. The component is also defined as insoluble or poorly soluble, resistant to proteolytic degradation.

There are multiple sources of amyloid fibrils (see Table 1). The most prevalent protein components of the fibril in CA are immunoglobulin light chains (AL) and amyloid transthyretin (ATTR). The latter can either be familial (ATTRv-CA) because of an inherited pathogenic variant of transthyretin (*TTR*) gene or non familial (ATTRwt-CA), caused by a wild type abnormal form of transthyretin, also known as senile cardiac amyloidosis. The other component of the fibrils are chaperone proteins of which the pentraxin serum amyloid P (SAP) is the most commonly represented.

**Table 1:** Classification of most common amyloid types with cardiac involvement.

Fibril Protein	Precursor Protein	Features	Other Target Organs
AL	Immunoglobulin light chain	S, L, A, H	All organs, usually except CNS
AH	Immunoglobulin heavy chain	S, L, A	All organs except CNS
AA	(Apo) Serum amyloid A	S, A	All organs except CNS
ATTRwt	Transthyretin, wild type	S, A	Heart mainly in males, ligaments, tenosynovium
ATTRv	Transthyretin, variants	S, H	PNS, ANS, heart, eye, leptomeninges
Ab2Mwt	b2-Microglobulin, wild type	S, A, HD	Musculoskeletal system
Ab2Mv	b2-Microglobulin, variants	S, H	ANS
AApoAI	Apolipoprotein AI variants	S, H	Liver, kidney, PNS, testis, larynx (C-terminal variants), skin
AANF	Atrial natriuretic factor	L, A	Cardiac atria

**Notes.**

S Systemic L Localized A Acquired H Hereditary HD Hemodialysis Associated CNS Central Nervous System PNS Peripheral Nervous System ANS Autonomic Nervous System

Modified from Sipe et al, Amyloid J 2016.^1^

It has been postulated that the main form of CA is the ATTRwt, though its real prevalence is not well established. More prevalent in men than in women (80% vs 20%) and with an age of onset that can be as early as mid sixties^2^, it is usually under-recognized as it can sometimes mimic other cardiac conditions that are found in the elderly population. Several data support overrepresentation of ATTRwt-CA. Series of autopsies have identified the deposit of TTR amyloid fibrils in up to 25% of postmortem hearts from individuals older than 80 years-old^3^*.* Recent studies also described a prevalence of 16% of patients with severe aortic stenosis undergoing transaortic valve replacement, condition which was associated with the low-flow low-gradient phenotype and mild reduced ejection fraction^4^. A French pilot study estimated that the prevalence of ATTRwt could be as high as about one third of patients with heart failure and preserved ejection fraction^5^. In spite of the recent increasing data suggesting the high prevalence of senile cardiac amyloidosis, this fact is not new, as it had been already reported by Pomerance in 1965, concluding that “*senile cardiac amyloid would appear to be the commonest existing form of amyloid, and likely to continue increasing with the increasing age of the population*”^6^.

The inherited form is less prevalent than the ATTRwt. The median age at diagnosis ranges from 60–70 years-old, depending on the gene variant. Over a 100 *TTR* gene pathogenic variants have been described, some endemic and ethnic-group mutations identified, with autosomal dominant pattern of inheritance. For instance, the p.Val122Ile pathogenic variant has been reported in African or Afro-Caribbean descent with an estimated population prevalence of 3% to 4%^7^*.* However, ATTRv-CA is not always found with isolated cardiac involvement, as it is mainly considered a neurologic disease, causing familial amyloidotic polyneuropathy.

From what respects to AL-CA, about 8-12 cases per million of prevalence is estimated. It results from any monoclonal gammopathy of hematologic disorders such as multiple myeloma (main cause), monoclonal gammopathy of uncertain significance or B cell dyscrasias. Among 30 to 50% of patients with AL amyloidosis have cardiac involvement^8^*.* The AL-CA mean age of presentation is 65 years-old, predominantly in female gender^9^.

There are other forms of cardiac amyloidosis, such as the secondary or reactive systemic amyloidosis, in which a chronic inflammatory state leads to an overproduction of the serum amyloid A (SAA) protein, a major acute-phase protein, that will eventually form the amyloid fibrils. Albeit, cardiac involvement is extremely rare and renal involvement dominates the clinical course in this patients^10^.

A particular and rare form of amyloidosis is the dialysis related amyloidosis, formed by the wild type deposit of Beta 2-microglobulin, a complication of long-term renal replacement therapy. Severe forms appear to manifest after 15 years of dialysis^11^.

#### 1.1.2 Etiopathogenesis

The predominant mechanisms of amyloid fibrils formations and the affinity for the tissue deposition differ depending on the amyloidosis syndrome. Even the causative effect of the organ dysfunction, although not well understood, may vary depending on the amyloid composition. For instance, in the heart, the deposition causes thickening of both ventricles, with subendocardial and diffuse distribution in the AL-CA whereas in ATTR can be a patchy or transmural involvement. It is also possible to find fibrils in the small intramural coronary arteries. The atrium, the conduction system, the valves and the pericardium can all be affected^12^.

**Figure 1.**
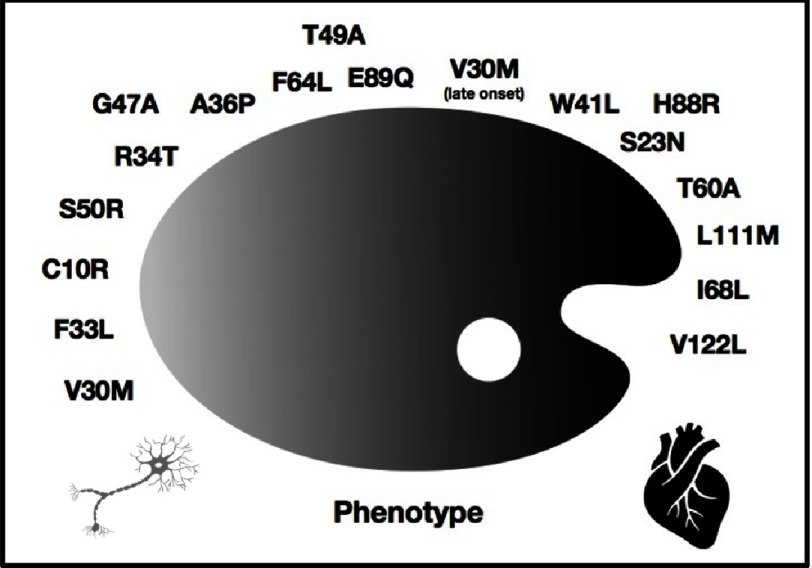
Spectrum of association genotype-phenotype in familiar ATTR, proposed by Rapezzi et al. From isolated peripheral neuropathy (V30M) to predominant cardiac phenotype (V122L) (modified from 13).

TTR is an homotetrameric protein that carries the thyroid hormone thyroxine (T4) and retinol-binding protein bound to retinol. It is mainly produced by the liver and in less quantity by the choroid plexus and retinal pigment epithelium. The dissociation of the protein into monomers, which is the rate-limiting step for amyloid formation, is the first point of discordance. Familial point mutations, usually missense variants that lead a change in protein amino acids, are known to destabilize the tetramer. Depending on the TTR mutation (Figure 1), the phenotype may change, from isolated neurological involvement to predominant cardiac features^13^. The explanation of the differential involvement is unknown. Val30Met (p.Val50Met renamed according to the updated nomenclature) is the most common mutation worldwide. It is endemic in some areas of Portugal, Japan, Sweden and Spain^14^. This variant has a bimodal presentation with an early onset disease in the third-to-fourth decade of life characterized by peripheral neuropathy and a onset leading to cardiac involvement^15^.

In the wild type syndrome, the gene is unaltered and the protein is then produced normally. The instability and dissociation of the tetramer a has been hypothesized to be caused by alterations in chaperone proteins^16^. The other fact that is required for amyloid fibrils formation is the aggregation of the monomers. The possibility of releasing aggregation-driving segment that were blocked in the tetrameric form is one plausible cause for the constitution of the cross–*β*-pleated sheet^17^.

In AL-CA, the free light chains (LC) produced by the clonal cells are less kinetically stable and more susceptible to unfolding. A faster unfolding of full-length chains activates the aberrant processing by endoproteases and interferes in the extracellular refolding, cutting the LC into segments that form aggregates for amyloid fibrils^18^. Similarly to what occurs with ATTRm, there is no explanation for the tissue tropism and the type of LC, but it has been described that regions IGVL1–44, IGLV2-14 and IGLV3-1 in the germline genes are related to AL-CA more often. This would support a genetic basis also in AL-CA^19^.

The mechanism of AA is the excess of production of SAA but, for unknown reasons, only a small proportion of patients with chronic inflammatory states develop amyloidosis^10^.

Eventually in all types, the amyloid fibrils act as cellular chemical stressors, producing reactive oxygen species, damaging the cells by mechanisms that are incompletely understood^20^*.* Myocyte dysfunction and apoptosis can also be induced by amyloidogenic light chains, directly and independent of independent of cardiac fibril formation through non-canonical activation of p38 MAPK signalling^21^.

**Figure 2.**
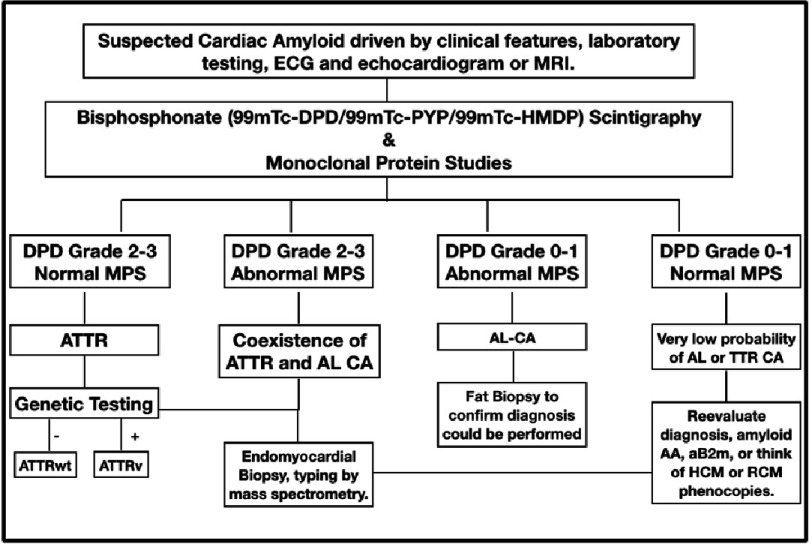
Diagnostic work-up algorithm. (DPD, (99m)Tc-3,3-diphosphono-1,2-propanodicarboxylic acid ((99m)Tc-DPD); MPS, monoclonal protein studies)^23^.

**Table 2:** Clinical Red Flags for CA syndromes.

**Figure 3.**
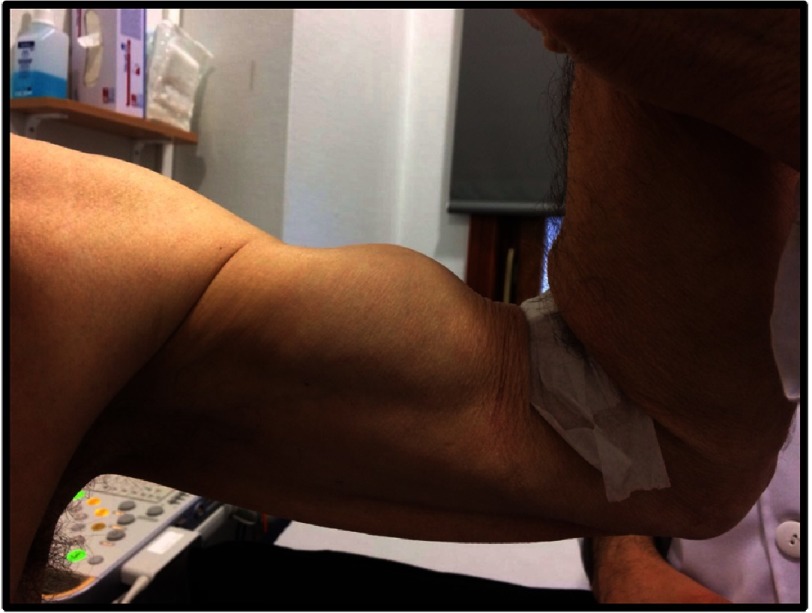
See the disproportionate prominence of contracted biceps, a result of distal biceps tendon non-traumatic rupture.

• Spinal stenosis (ATTRv-CA)
• Symmetric sensorimotor peripheral neuropathy in lower extremity (ATTRv-CA)
• Postural hypotension and orthostatic syncope.
• Gastric emptying disorder, pseudo-obstruction.
• Chronic diarrhea and weight loss.
• Erectile dysfunction.
• Macroglossia and periorbital purpura (pathognomonic in AL-CA)
• Easy bruising without anticoagulation.
• “Popeye” sign or deformity of the biceps muscle, after tendon non traumatic rupture (Figure 3).
• Vitreous opacities, cotton wool type (ATTR)
• Bilateral Carpal tunnel syndrome (ATTR)

#### 1.1.3 Diagnostic work-up

The diagnosis of CA can be challenging. The diffuse deposition and organ involvement, together with the nonspecific signs and symptoms, makes the diagnostic work-up complex and the need for the clinician to be aware of the right clues, to reach diagnosis without time delay. Usually, patients may be visiting more than one physician before diagnosis is reached^22^.

The diagnostic work-up should be initially based on clinical features, electrocardiogram and echocardiogram. Then, if the suspicious of CA is high, complementary tests will be performed, if feasible, starting from the non-invasive ones (Figure 2).

##### 1.1.3.1 Clinical features and family history.

Fatigue is the main clinical symptom due to diastolic dysfunction. Hypertrophic cardiomyopathy or hypertensive cardiomyopathy can rise as an erroneous diagnosis in these patients. Palpitations related to the novo atrial fibrillation are another referral reason. Congestive heart failure or low cardiac output state, associated with asthenia, cardiac cachexia and orthostatic hypotension, are end-stage disease manifestations. Usually, renal failure exacerbates the clinical picture and the refractoriness to heart failure therapy (Table 2).

Particularly in AL amyloidosis, it is rare to have the diagnosis before symptoms appear and, when this happens, the patients may already have advanced organ damage. Typical findings include neuropathy, nephrotic syndrome, hepatomegaly, peri-orbital bleeding and macroglossia. Some of these findings are also shared with ATTR-CA, i.e., neuropathy or hepatomegaly caused by congestive heart failure or chronic kidney disease^24^.

##### 1.1.3.2 Electrocardiogram (ECG).

There are almost always pathologic findings in the ECG (Table 3). Some registries report less than 2% patients with normal ECG. The most reliable electrocardiographic characteristic to support CA diagnosis is the relation between the left ventricular wall thickness and the QRS voltage, first described by Carrol in 1982^25^. A simpler index was latter proposed by Quarta et al., considering the division of total QRS score (the arithmetic sum of the QRS complexes in peripheral and precordial leads) by LV wall thickness^26^.

**Table 3:** Electrocardiographic findings from refs 9, 26–28.

• Low-voltage criteria: QRS amplitude less than 5 mm in limb leads or less than 10 mm in precordial leads. (About half of AL-CA patients and about 30% of ATTR-CA.)
• Pseudoinfarct pattern (About 30% to 60% of the patients).
• Atrial fibrillation (varies according to etiology with a mean value of 15% to a prevalence of 40% in ATTRwt amyloidosis).
• QRS duration (ms) in ATTR-CA is higher than in AL-CA (122.3 ± 34.9 vs 103.4 ± 25.6). In about 30% of the patients the QRS duration is over 120 ms.
• Any degree of AV-block is found in about 15% to 30% of patients.

The study of syncope is particularly complex in these patients as there is a tendency to hypotension resulting from both dysautonomic disorders, low output and diuretics and bradyarrhythmias. About 20% of ATTRwt patients need a pacemaker and it has been suggested that prophylactic pacemakers should be implanted in some scenarios^29^.

##### 1.1.3.3 Laboratory Tests.

1.1.3.3.1 Monoclonal Protein Studies (MPS)

MPS is mandatory to rule-out AL-CA. Blood serum electrophoresis is less accurate than immunofixation (IFE) of serum and urine to demonstrate a monoclonal band and, quantification of serum free light chains (sFLC), is used to obtain the kappa/lambda ratio, which is abnormal in >90% of patients with untreated AL amyloidosis. The definition of abnormal FLC ratio is <0.26 or >1.65 (slightly variations depending on laboratory assays)^30^. The combination of serum and urine IFE and quantification of serum free light chains (sFLC) have a 99% sensitivity for identifying AL-CA substrate. However, two caveats in this studies merit discussion. The first, is the high prevalence of monoclonal gammopathy of uncertain significance (MGUS) in patients older than 75 years-old, which makes difficulties in distinguishing between ATTRwt-CA and AL-CA in this population. A moderate increase in circulating free light chains is not necessarily pathological in this subgroup^31^. The second is the changes in clearance of the FLC depending on renal function, where the reference range switches from 0.26 to 1.65 (normal renal function) to 0.37 to 3.1 (renal failure)^32^.

 1.1.3.3.2 NT-pro-BNP, Troponin and Proteinuria

Troponin and natriuretic peptides levels are usually high in CA due to LV dysfunction and the direct toxicity of amyloid fibrils and precursors. This is particularly important in AL-CA^33^. Proteinuria with or without impaired glomerular filtration rate should raise suspicion as well.

##### 1.1.3.4 Echocardiography.

**Figure 4.**
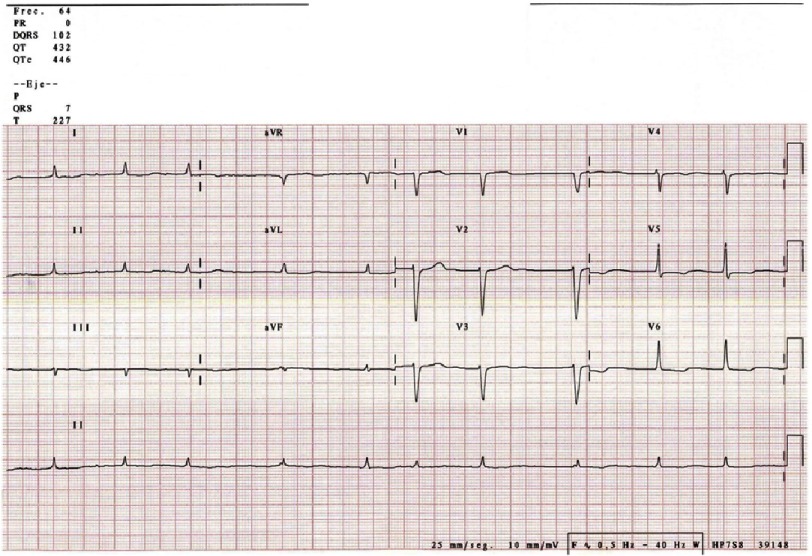
Typical electrocardiographic findings, showing atrial fibrillation, low voltages and pseudoinfarct pattern. Male patient, 86 years-old.

**Figure 5.**
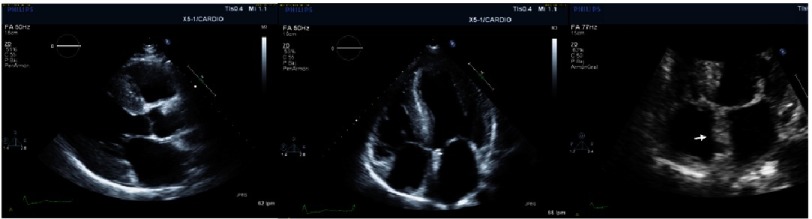
2-dimensional echocardiographic images of a patient with ATTR-CA. Concentric LVH is visible in the first on the left and in the image in the middle. Ground glass appearance of the myocardium is also apparent. In the image on the right hand side we can appreciate interventricular septum thickening (white arrow) and valve thickening.

**Figure 6.**
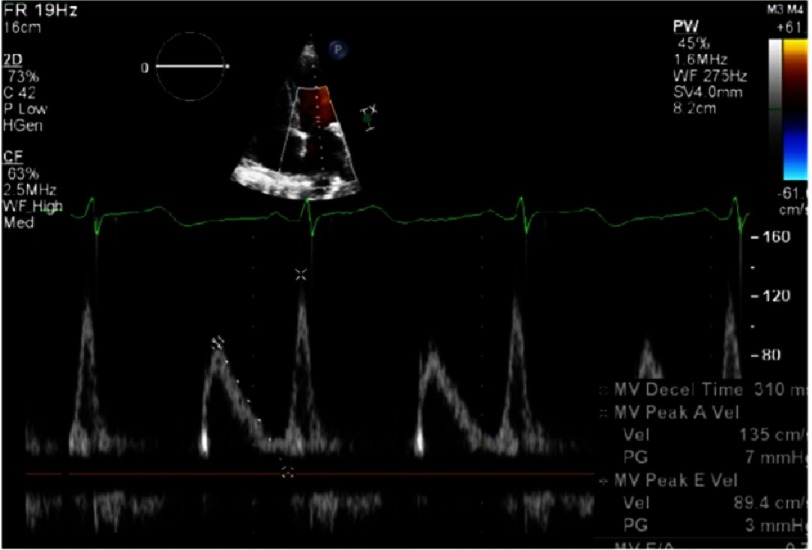
Diastolic dysfunction in early stages, shown by E/A relation <1 in Doppler wave mitral inflow velocities.

**Figure 7.**
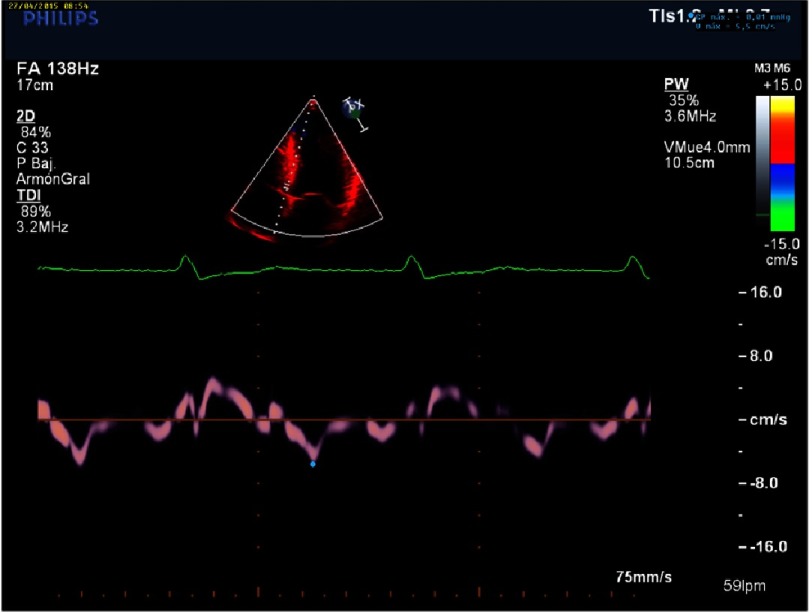
Low velocities in tissue Doppler imaging of the mitral annulus (under 8 cm/s) can be seen in early stages.

**Figure 8.**
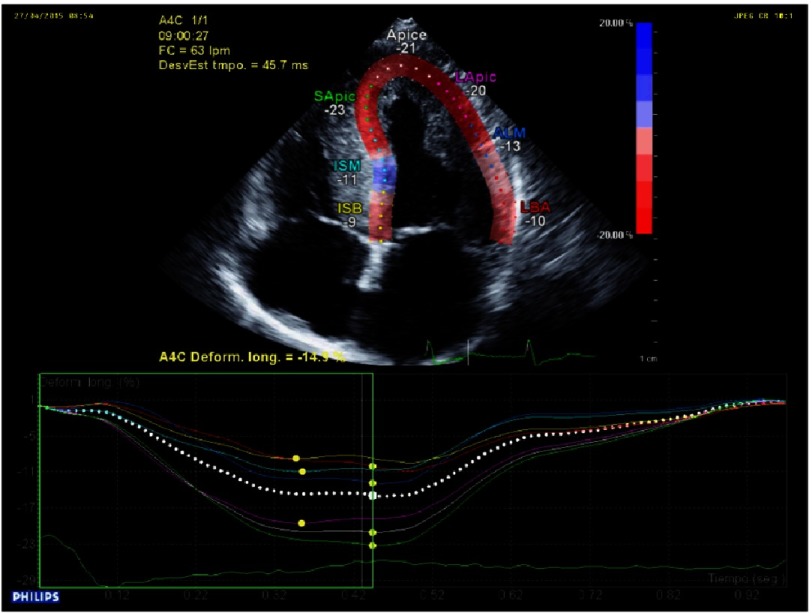
Abnormal ventricular strain and strain rate, typically with apical sparing. Apical Strain/ Basal Strain >2.1.

**Figure 9.**
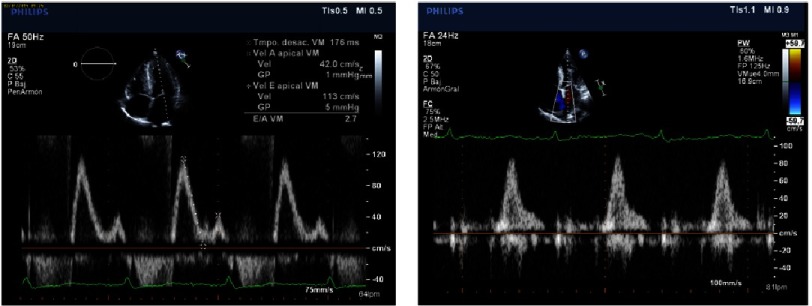
Restrictive physiology. In the image on the left, E wave high velocities measured in the mitral inflow with very low A wave velocities with abnormal deceleration time, indicates high left atrial pressure and poor compliant left ventricle. On the right hand side, the pulmonary vein Doppler velocities show absence of systolic inflow wave and high diastolic velocities, consistent with restrictive physiology as well.

An increase in left ventricle thickness is the most obvious imaging sign of CA (Figure 5). Restrictive physiology (Figure 8) may not be present early in the disease and ejection fraction is usually in the low range of normality, although the ventricle function is abnormal (Figures 4–9) (Table 4).

##### 1.1.3.5 Bisphosphonate (99mTc-DPD/99mTc-PYP/99mTc-HMDP) scintigraphy.

**Figure 10.**
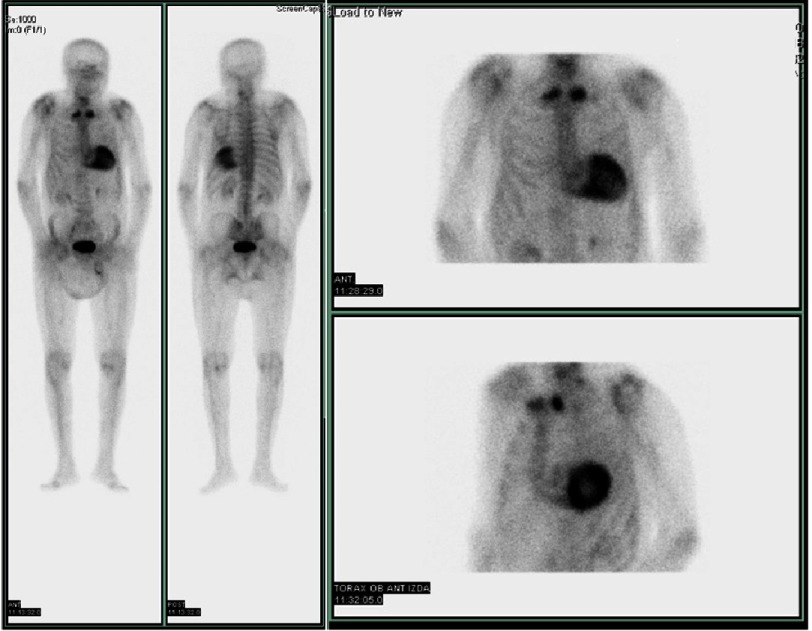
Bisphosphonate scintigraphy. Grade 3 uptake score in a patient with ATTR-CA.

Bone scintigraphy using (99m)Tc-3,3-diphosphono-1,2-propanodicarboxylic acid ((99m)Tc-DPD) or similar has become the gold standard for the diagnosis of TTR related cardiac amyloidosis with a specificity and positive predictive value of 100% when grade 2 or 3 myocardial radiotracer uptake on bone scintigraphy was found, in absence of a monoclonal protein in serum or urine^23^*.* The semiquantitative visual score of cardiac retention, graded from 0 to 3, compares the uptake of the heart with the bone, being heart grade 2 uptake equal to bone (Figure 10).

**Table 4:** Echocardiographic clues in HCM phenocopies (storage disorders)^34^.

• Increased interatrial septum thickness (Amyloidosis)
• Increased atrioventricular valve thickness (Amyloidosis; Anderson–Fabry disease)
• Abnormal ventricular strain and strain rate, typically with apical sparing (Amyloidosis) (Figure 7).
• Increased RV free wall thickness (Amyloidosis, Anderson–Fabry disease)
• Mild–moderate pericardial effusion (Amyloidosis)
• Ground-glass appearance of ventricular myocardium (Amyloidosis)
• Concentric LVH (Amyloidosis, Glycogenosis, Anderson–Fabry disease)
• Extreme concentric (LVH Danon disease, Pompe disease)
• Global hypokinesia (with/without LV dilatation) Anderson–Fabry; mitochondrial disease; TTR-related amyloidosis; PRKAG2 mutations; Danon disease)

##### 1.1.3.6 Cardiac Magnetic Resonance Imaging (CMRI).

Cine CMRI imaging permits quantification of ventricular ejection fraction and chamber volumes, with great accuracy to measure and characterize ventricular hypertrophy and its distribution. In this line, asymmetrical LVH was described recently to be the commonest pattern among ATTRwt-CA^35^ based on MRI studies. The LGE distribution pattern in the LV can be variable and corresponds to underlying fibrosis and amyloid deposition. LGE can be global subendocardial (the typical one), transmural, localized or patchy. Right ventricular and left atrial LGE often appear. One particular distinction in cardiac amyloidosis assessment by CMRI is that achieving good quality acquisitions and interpretation of the LGE is really challenging due to the difficulties in the election of the optimal inversion recovery time. This is a source of false negative results that might be reduced with the implementation of automatic tools like the PSIR (phase sensitive inversion recovery) sequence. Assessment of the myocardial T1 relaxation times with T1 mapping can overcome this problem^35^.

##### 1.1.3.7 Biopsy.

Endomyocardial biopsy (EMB) is considered the gold standard for diagnosis of storage diseases^36^. However, the variable distribution of amyloid in the heart leads to false negative biopsies, as some parts of the myocardium do not contain the deposits. Peri-cellular and nodular interstitial involvement are the most common patterns although less frequently, deposits in the arterial wall are also seen. In systemic amyloidosis biopsies can be taken from abdominal fat, rectal mucosa, oral cavity mucosa or minor labial gland. Congo red and and Sirius red, are the histological staining used, showing apple-green birefringence when viewed with cross-polarising filters in a light microscope. Electron microscopy visualises the form (non-branching) and size of the fibrils (7–10 nm in diameter). Typing the amyloid fibril is also possible by using immunohistochemical examination (IHC) or mass spectrometry-based (MS) methods.

##### 1.1.3.8 Genetic testing.

Genetic testing, whenever possible, is recommended for differentiating ATTRwt from the familial form of ATTR, with the corresponding genetic counseling for the family members^34^.

#### 1.1.4 Staging and prognosis

##### 1.1.4.1 ATTR-CA.

In 2017, Gillmore et al. proposed a new staging system for ATTR-CA with the NT-proBNP peptide and the estimated glomerular filtration rate. Stage I was defined as NT-proBNP ≤3000 ng/L and eGFR ≥ 45ml/min, Stage III was defined as NT-proBNP >3000 ng/L and eGFR <45 ml/min, and Stage II, the remainder. Among the cohort that they used to validate the system, the median survival for stage I, II and III was 69.2months, 46.7 months and 24.1 months respectively^37^. A recent study reported that lower baseline serum TTR concentration levels is independently associated with poor outcomes in this patients in patients with ATTRwt^38^.

##### 1.1.4.2 AL-CA.

The staging system for AL-CA proposed in 2012 by Kumar et al. (Mayo Clinic, Rochester, MN) is based on FLC-diff (difference between involved and uninvolved light chain), cTnT (cardiac troponin T biomarker), and NT-ProBNP. Patients were assigned a score of 1 for each of FLC-diff >18 mg/dL, cTnT >0.025 ng/mL, and NT-ProBNP >1,800 pg/mL. Stages I to IV were created and the median overall survival from diagnosis was 94.1, 40.3, 14, and 5.8 months, respectively^39^.

#### 1.1.5 Treatment

The severity of the disease at diagnosis determines the response to the treatment. Central to new treatments in CA is the reduction of precursor proteins that form amyloid fibrils.

##### 1.1.5.1 Amyloid non-specific therapy.

Maintenance of fluid and electrolyte balance is crucial for heart failure management. The usual drug therapy for heart failure not only has not demonstrated benefits in CA but has also be seen to be harmful. In particular, vasodilators can exacerbate orthostatic hypotension and syncope and Beta-blockers can cause severe bradycardia. There is a high risk of toxicity with digoxin and nondihydropyridine calcium channel blockers caused by their high avidity to amyloid fibrils^40^.

Heart transplant for very selected patients can be done, but is controversial. Restrictive selection of recipients and adequate management of underlying disease in AL-CA or ATTRv with modern therapies may improve outcomes^41^. Inflow cannula “suck-down” events, arrhythmias and septum displacement causing impaired right ventricle function are potential problems with ventricular assis devices^42^.

##### 1.1.5.2 Amyloid specific therapy.

Diflunisal, a nonsteroidal anti-inflammatory, has been shown to improve neurological symptoms and Tafamidis has been demonstrated to delay disease progression^43^ in familiar peripheral neuropathy caused by *TTRv*. Ongoing trials are evaluating the effect on the heart of Tafamidis^44^. Only liver transplant for ATTRv-CA and treatment of hematologic monoclonal disorder, in AL-CA, is currently approved. Albeit, there are promising molecules coming in the near future (Table 5). In example, antibody therapies such as the one targeting residues from 115–124 of the *Met30Val* variant has been tested with encouraging results^45^.

**Table 5:** Potential therapies in Cardiac Amyloidosis.

• Synthesis suppression:
∘ Eliminate the clonal plasma cells and stem cell transplantation (AL-CA)
∘ Liver transplant (ATTRv)
∘ Epigallocatechin gallate (Green Tea). (ATTR)
∘ Patisiran (small interfering RNA, reduces transcription of TTR)
∘ Ionis (interference with the splicing of TTR)
• Stabilizers (prevent the homotetramer of TTR from dissociation into monomers):
∘ Diflunisal (ATTRv)
∘ Tafamidis (ATTRv)
∘ Doxiciclina (ATTR)
∘ Tolcapone (ATTR)
∘ AG-10 (selective to ATTRwt and ATTRv- *V122I*)
• Deposit removal (clearance):
∘ The antiamyloid 11-1F4 monoclonal antibodies
∘ Pyrrolidine-2-carboxylic acid + IgG anti-SAP antibodies (ATTR, AL)
∘ Antiamyloid monoclonal NEOD001

### 1.2 Cardiac oxalosis (CO)

CO is an extremely rare cause for LVH, consisting of extracellular deposit of oxalate, due to an overproduction caused by a deficiency of the alanine-glyoxylate aminotransferase liver enzyme. The underlying diseases are named primary hyperoxalurias, rare autosomal recessive disorders of oxalate metabolism. Heart failure as a result of systolic dysfunction and restrictive cardiomyopathy, with LVH in one third of the patients, together with rhythm disorders, are the cardiac features found in this entity. Urolithiasis, nephrocalcinosis and progressive renal failure, complete the rest of this syndrome’s picture^46^.

## 2 Intracellular deposit storage diseases

There are more than 50 lysosomal storage disorders described^47^. Those most commonly associated with hypertrophic cardiomyopathy phenotype are Fabry disease, Pompe disease and Danon disease. Although previously associated to lysosomal disease, because increased deposits of glycogen and amylopectin occurs in the lysosome, PRKAG2 syndrome’s etiopathogenesis cornerstone is the AMP kinase dysregulation, a non-lysosomal-associated enzyme. Mucopolysaccharidoses (MPS) type IH and II and the classical glycogen storage diseases that produce hypertrophy in the heart (types II, IIa, III and IV) will also be discussed below.

In the initial evaluation of a patient with the suspicion of a metabolic disorder, some general investigations should be performed (Table 6). It is important to rule out growing-up disorders in the infants, by plotting growth centiles. Dysmorphic appearance or skin abnormalities should be assessed and a neuro-ophthalmological examination is mandatory. Respiratory or liver involvement of the disease is also part of the work-up. Clinicians should be aware of the complexity of some specialized biochemical tests that may require special collection methodology. Storage of DNA for pathogenic variants analysis is particularly useful, in addition to the study of family history. In some cases might be of utility to take a skin biopsy for cell culture in order to perform enzyme tests.

**Table 6:** Useful laboratory tests^48^.

General tests:	Glucose, Urea, Electrolytes, Creatinine, Liver function tests. Urine ketones.
	Blood gases, ammonia, lactate, anion gap.
	Full blood count.
Specific tests	Carnitine, acylcarnitine profile, CSF lactate and amino acids.
	Urine organic acids, urine amino acids, plasma amino acids, uric acid

In the differential diagnosis of LVH, the ECG is an essential test and there are hints that suggest a possible metabolic disorder . For example, ventricular pre-excitation may be seem in PRKAG2 syndrome, Danon disease, Pompe disease as well as other non storage disorders like MELAS syndrome, Leigh syndrome, MERRF syndrome or oncocytic cardiomyopathy.

### 2.1 Mucopolysaccharidoses (MPS)

Hurler syndrome (MPS type IH, autosomal recessive) and Hunter syndrome (MPS type II, X-linked) are the two MPS that may provoke myocardial thickening. Other cardiovascular abnormalities associated are valvular disease, coronary artery narrowing or pulmonary and systemic hypertension.

### 2.2 Anderson–Fabry disease

#### 2.2.1 Introduction

Fabry disease (Anderson–Fabry Disease (AFD) or *angiokeratoma corporis diffusum*) is a rare disorder of the metabolism of the *α*-galactosidase A, a lysosomal enzyme that degrades neutral glycosphingolipids, mainly globotriaosylceramide (Gb3), resulting in progressive intracellular accumulation.

#### 2.2.2 Pathogenesis

*GLA* is the gene that encodes *α*-galactosidase A and maps to Xq22.1. Variants in this gene can affect enzyme synthesis, folding, degradation, trafficking or activity, leading to inability to degrade Gb3. Males have low or absent enzyme activity, but as a result of the lyonization process (transcriptional inactivation of one of the X chromosomes in some cells during embryogenesis) may present with atypical forms and a wide spectrum of signs and symptoms (Figure 12), depending on enzyme’s activity preservation or absence among the different organs and tissues^49^.

#### 2.2.3 Diagnostic work-up

##### 2.2.3.1 Clinical presentation

2.2.3.1.1 Cardiovascular manifestations

Heart disease is present in all forms of AFD, with concentric LVH as the hallmark. The proportion of patients with Fabry disease among those previously diagnosed of HCM, ranges from 0.5% to 12% depending on the reported series^50^.

**Figure 11.**
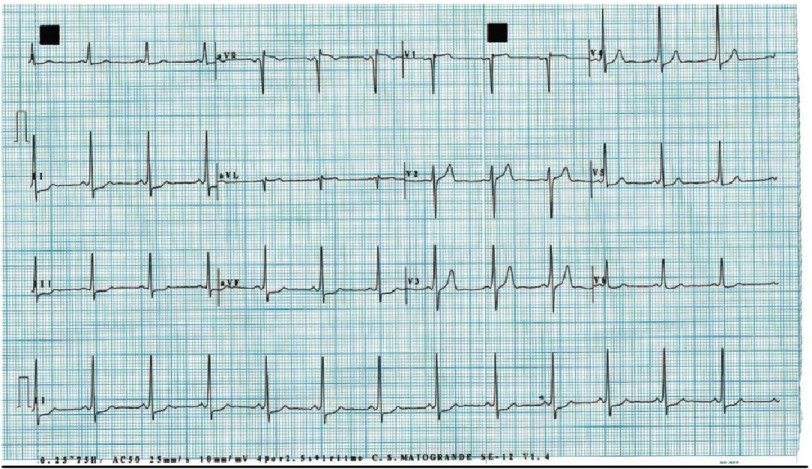
Electrocardiogram of a 20 year-old male patient with classic form of AFD. Sinus rhythm and short PR interval is patent. Repolarization abnormalities with T wave inversion in V3 to V6 and I an AVL is also present.

The electrocardiogram in AFD is a very useful tool as a diagnostic red flag (LVH criteria and short PR interval) and for risk stratification, as atrial fibrillation and conduction system disease, mainly complete atrioventricular block (Figure 14). Short PR interval (Figure 11), which can be present in children under 10 years-old^52^, is seen in about 40% of the patients^53^. Interestingly, it is not due to an accessory pathway but to an accelerated conduction of the atrioventricular node (AVN). Some studies report 17% incidence of AF on ambulatory monitoring. Also ventricular arrhythmias driven by fibrosis and conduction abnormalities can be present in 8% of cases^54^.

Sudden cardiac death, though rare, can be the first presentation, due to ventricular arrhythmias or complete AV block. Heart valves can be affected in about 15% of the patients, with thickening, fibrosis and calcification, but usually does not result in significant impairment^51^.

Some AFD patients complain of angina. This is more commonly seen in combination with LVH and might be due to increased oxygen demand for similar reason to what occurs in HCM, but also in response to diffuse arteriopathy associated to cellular damage of the arterial walls. The prevalence of aortic dilatation (AD) increases with age and it is more prevalent in male and usually affects the sinus of Valsalva and the ascending aorta^55^. It was reported a AD prevalence of 9,6% in males by Barbey et al.^56^.

Hypertension is also overrepresented (about 50%), contributing to increase cardiovascular risk and ventricular hypertrophy.

Echocardiography is essential in the diagnostic work-up, staging and follow-up. Table 7 describes some of the typical findings.

**Figure 12.**
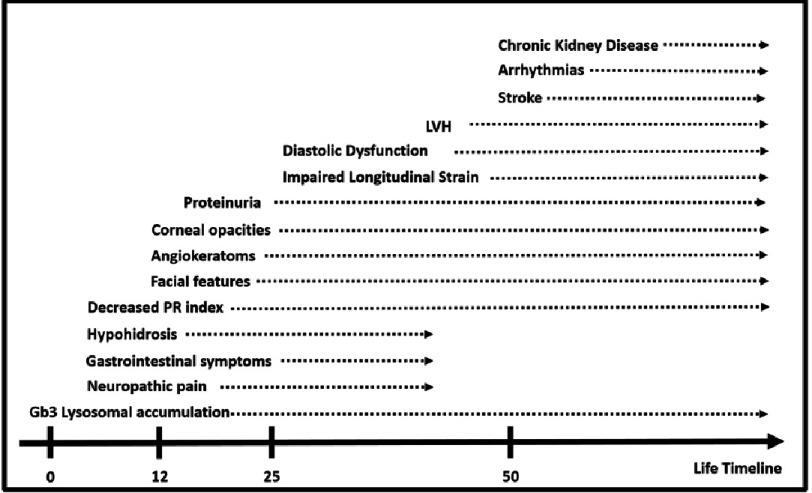
Red Flags and life-time relation. The chart shows the approximate onset of the symptoms and duration. Hypohidrosis, gastrointestinal symptoms and neuropathic pain, in some patients may disappear with age.

**Table 7:** Summary of echocardiographic findings^57^.

Left Ventricle	Increased wall thickness: concentric (not exclusively but most common). Mean age: 42.0 ± 14.5 years in men and 50.1 ± 12.0 years in women
	Binary sign, hyperechogenic endocardial border adjacent to a hypoechogenic subendocardial layer. Sensitivity 15.4% - to 28% and a Specificity of 73.3% to 80%.
	Thickening and hyperechogenicity of papillary muscle
	Ejection fraction (EF) of LV generally preserved in early stages. Diastolic function often impaired. Reduced systolic longitudinal strain in basal and posterolateral segments. Loss of base-to-apex circumferential strain gradient.
Right ventricle	RVH. EF and Function behaviours similar to LV.
Atria	Mild to moderate enlargement
Valves	Mitral valve prolapse and leaflet thickening (also aortic) and redundancy.
Aorta	Sinus of Valsalva and ascending aorta dilatation (not the descending)

Significant LVOT obstruction has been described in very few cases, although Calcagnino et al. reported in 2011 a series of 14 symptomatic patients, of which 6 had provoked LVOT significant gradient^58^.

LV ejection fraction is generally preserved but in advanced disease it can be reduced, terminating in end stage heart failure. Diastolic function is impaired but restrictive physiology is rare, unlike other infiltrative disorders like CA. For both diastolic and systolic function, some studies have seen that impairment begins with the increased wall thickness or fibrosis^59^. Fibrosis is assessed in the MRI with LGE, being the typical finding the localized enhancement of the basal posterior left ventricle wall, which can be accompanied by wall thinning in late stages. Notwithstanding, more sensitive techniques like strain or MRI have shown markers of disease earlier than the ordinary echocardiographic findings. Global systolic longitudinal strain is lowered in patients with Fabry disease without LVH. Based on this statement, and to facilitate natural history description and variations with potential treatments, Yeung et al. have proposed a revised (from Weidemann et al.) stage classification as in Table 8:

**Table 8:** Stages in Fabry diseases based on MRI and echo-strain^57^.

Stage	LGE	LVH	Longitudinal Function	Radial Function
0	No	No	Normal	Normal
I	No	No	Reduced	Normal
II	No	Yes	Severely reduced	Reduced
	Yes	No	Severely reduced	Reduced
III	Yes	Yes	Severely reduced	Severely reduced

Since the advent of new techniques in MRI such us T1 mapping (shortened in AFD patients in early stages), new studies are in progress and will definitely help to better understanding of disease history and treatment response^60^. Focal 18F-FDG uptake has been demonstrated in some patients and is associated with impaired left ventricular longitudinal function^61^*.*

2.2.3.1.2 Kidney disease

Renal impairment is preceded and caused by de deposit of Gb3 in podocytes, endothelial, epithelial, and tubular cells. Proteinuria is the first manifestation, leading to end-stage renal failure without treatment in the fifth decade of life. It has been reported that podocyturia might be an earlier marker of disease than proteinuria, although microalbuminuria/proteinuria is the most commonly used method for kidney function assessment in early stages (first or second decade of life)^62^. Novel procedures to differentiate female AFD patients are proteomic analysis or urinary Gb3 or lyso-Gb3 determination^63^.

 2.2.3.1.3 Neurological disease

Peripheral neuropathy often manifests as pain crisis more than as acroparesthesias. Hearing loss is common and it is progressive. Hypohidrosis happens in 53% of males and 28% of females, and the age at onset is 23 ±  17 years in males and 26 ±  20 years in females, according to the FOS (Fabry Outcome Survey, Orteu et al. 2007). Both neuropathic pain and hypohidrosis are associated with heat intolerance. Ischemic stroke, weather due to vasculopathy or to atrial arrhythmias, may present in the fifth decade of life. Recent research suggests that there may be an increased risk of developing Parkinson Disease in individuals with *GLA* mutations^64^.

2.2.3.1.4 Gastrointestinal

Autonomic nervous system dysfunction, vasculopathy and tissue inflammation are the main proposed mechanisms of the ethiopathogenesis of gastrointestinal disturbances in AFD. All this may translate into abdominal pain, intestinal and stomach motility disorders, malabsorption, etc. In children it impacts on quality of life, academics and plotting growth centiles. The psychological stress is also a feedback to bolster gastrointestinal symptoms^65^.

2.2.3.1.5 Dermatological

The most common finding are the angiokeratomas, reported in 66% of males and 36% of females, though is not specific of AFD. Whereas in men are usually located in what it is called the “bathing trunk” area, in women it is more likely to find them on the proximal limbs and the trunk. The other cutaneous symptom related are telangiectasias on face, lips, mucosa and areas exposed to the sun like the V of the neck^66^. Lower limb oedema and lymphoedema may be also patent. Typical facial features in the classic form can be in up to 50% of the patients, showing prominent earlobes, lips and nasolabial pledges or heavy hairy eyebrows^67^.

2.2.3.1.6 Ophthalmological

Visual impairment is an uncommon but slit-lamp evaluation will show the cornea verticillata (CV), which respond to golden brown or gray deposits in the inferior interpalpebral portion of the cornea in a clockwise fashion, is the most common finding. Amiodarone, chloroquine or indomethacin can also cause CV. CV can be present in females as a milder form. Another site of deposits is the posterior capsule, causing the Fabry cataract. Some patients also exhibit aneurysmal dilatation and tortuosity of conjunctival and retinal vessels, sign that has been proposed to be a new sensitive prognostic marker^68^.

2.2.3.1.7 Others

Pulmonary involvement (chronic bronchitis, wheezing, or dyspnea), skeletal (osteopenia and osteoporosis) and lymphatic (limb lymphedema and pitting edema) has also been described in some patients.

##### 2.2.3.2 Diagnostic Tests

2.2.3.2.1 Enzyme assay

**Figure 13.**
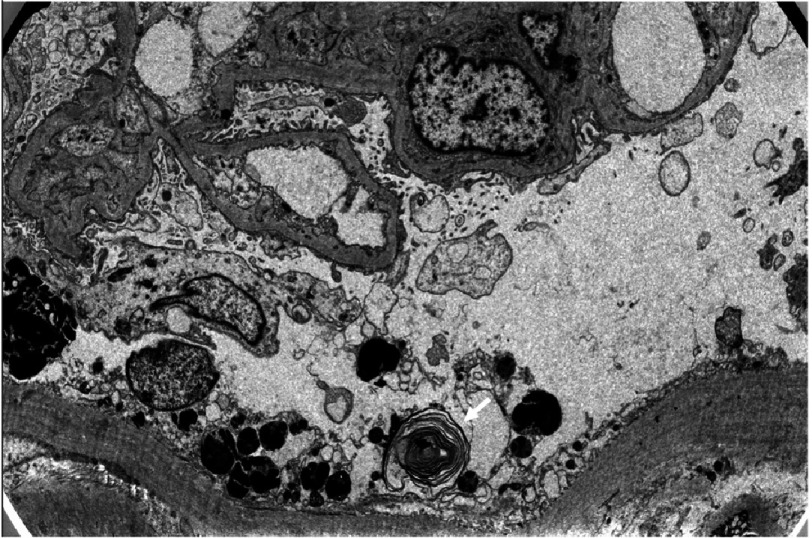
Renal biopsy under electron microscopy showing the typical zebra body, formed by Gb3 deposit in the lysosome (white arrow).

Alpha-galactosidase (*α*-GLA ) activity assay alone is diagnostic when it is absent or very low in men, although genetic testing to look for pathogenic variants is highly recommended, because in some cases benign polymorphisms can cause reduced levels of the enzyme. The easiest way to look for the enzyme activity is to go for the dried blood spots test. Leukocyte enzyme can also be tested and it is useful in atypical forms of AFD. In women, as they can still have some activity, a disease-causing mutation should be the target for making the diagnosis. For uncertain significance variants, if available, in vitro GLA mutation expression assays would be the definite test. Albeit, since is not widely used, a consistent co-segregation study would fulfil the criteria requirements for diagnosis establishment. In some cases, mostly atypical variants, biopsy can make the diagnosis (Figure 13). Although renal biopsy’s yield is higher, starting from a skin biopsy could be less invasive.

**Figure 14.**
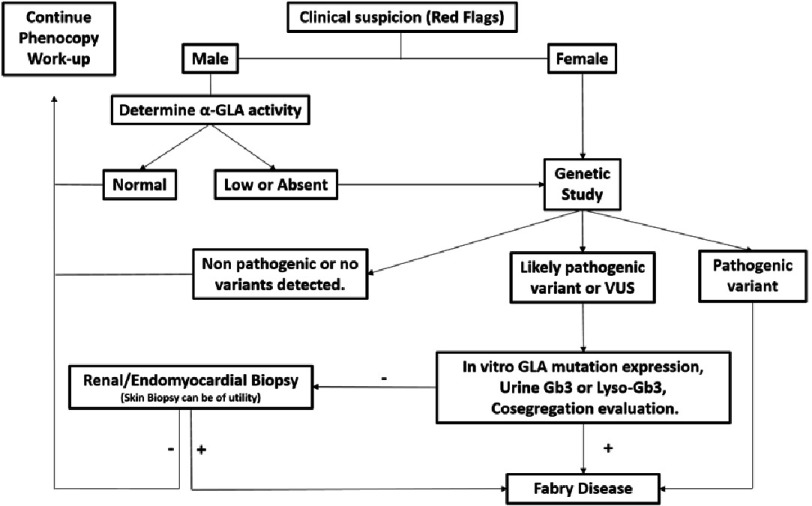
Algorithm for diagnostic work-up.

Apart from testing the *α*-GLA activity, it is also possible to get classic form of AFD diagnosis if the urine Gb3 determination is in the high range and for those with atypical form of AFD, determination of lyso-Gb3 in urine is also a diagnostic useful tool^69, 70^.

2.2.3.2.2 Genetic testing

The majority of the causal pathogenic variants in AFD exhibit a predilection to nonsense insertion/deletion or premature truncation mutations because of a frameshift. Whereas missense mutations appear to be less prone to cause disease, it has also been suggested that some missense variants can be cause of late onset forms or in some cases are associated with particular phenotypes. This is the case of the p.N215S pathogenic variant, which presents with cardiomyopathy in the absence of renal involvement^71^. Disease expression may vary among members of the same family, even between males, which makes treatment decisions and counselling more difficult.

Implications of genetic study not only have to do with diagnosis or counselling but also with treatment, since some specific treatments are solely effective against certain pathogenic variants. Variants of uncertain significance should be cautiously evaluated, and an upgrade in pathogenicity degree ought be carefully proved by functional studies or a consistent co-segregation analysis, following guidelines consensus^72, 73^. Urine Gb3 or lyso-Gb3 can be useful in this scenario.

##### 2.2.4 Life expectancy and cause of death

According to data from the Fabry Registry, life expectancy in USA for men was 58.2 years (74.7 years in men population) and 75.4 years for women (80.0 years in women population). The main cause of death varied in the registry, being renal failure for deaths before 2001 and cardiac disease for the patients who died after that period. These may be biased by the effect of ERT, although it has not been proved. Also dialysis and ACEI/ARB adjunctive therapies are related to improved outcomes.

All this is general AFD data population and each individual can have a different surveillance scenario, mostly depending on the genetic GLA variant and expressivity.

##### 2.2.5 Management

Non-specific treatment includes angiotensin converting enzyme inhibitors (ACEIs) or angiotensin receptor blockers (ARBs) to maintain a urine protein to creatinine ratio less than 0,5 g/g. Some evidence also support the potential use of medications such as amiloride as podocyte stabilizator and antiproteinuric or paricalcitol^63^.

Active investigation for atrial fibrillation is recommended and anticoagulation should be initiated independently of CHADSVASC score. No stroke primary prevention strategy with antiplatelet therapy or lipid-lowering agents has shown any current evidence, although individualized decision is reasonable.

Pain release is a mayor issue to solve, and neuroleptics drugs are an option for neuropathic pain (gabapentin, carbamazepine). For pain crisis, opioids can be used.

Heart or renal transplant is feasible at end-stage organ burden, considering that the *α*-GLA activity is normal in the donor and the receptor will be free from Gb3 deposit in the transplanted organ.

###### 2.2.5.1 Enzyme replacement therapy (ERT).

There are two available ERT, intravenously administered. The agalsidase beta, with a posology of 1 mg/kg every 2 weeks (European Medical Agency (EMA) and US Food and Drug Administration (FDA) approved) and agalsidase alfa, with posology of 0.2 mg/kg every 2 weeks (EMA approved).

There is not a complete consensus for ERT initiation indications, and symptoms or organ manifestations criteria may vary among the different guidelines. Also the level of evidence is often driven by expert consensus or small RCT. Following the recommendations of USA Expert Panel 2016, ERT should be started in the classic form of AFD in symptomatic children at any age and in asymptomatic males with classical mutations by age of 8–10 years. For asymptomatic females, decision should be made considering family history of females. This patients should be followed closely to reassess therapy initiation^74^. In adult patients, starting ERT should be considered at the time of diagnosis, although in end-stages it may not be effective^75^. In patients with late onset form o atypical form of AFD, organ damage (laboratory biomarkers, biopsy or echocardiogram) evidence is required to support the initiation of the ERT.

###### 2.2.5.2 Non-ERT approaches.

Recently, the European and Canadian Medical Agencies have approved migalastat, an oral medication consisting of a molecular chaperone. Chaperones are proteins that assist the covalent folding or unfolding and the assembly or disassembly of other macromolecular structures. For this reason, migalastat is only useful for certain pathogenic variants of GLA.

##### 2.2.6 Screening recommendations

First-degree relatives should be evaluated, for early diagnosis purposes, co-segregation analysis and counselling. Concerning newborn screening, it is not currently included in most of the government’s screening programs, although some centers have started doing it. Newborn screening is challenging, as it is the management and counselling of possible AFD detected cases. The phenotype and prognostic heterogeneity of the disease, together with the unknown significance of many rare variants, can make newborn screening a double-edged sword.

### 2.3 Glycogen storage diseases

The classical glycogen storage diseases that produce hypertrophy in the heart are types II, IIa, III and IV, and will be discussed below.

#### 2.3.1 Glycogen storage disease type II (Pompe disease/acid alpha-glucosidase deficiency/acid maltase deficiency)

The first identified lysosomal storage disorder was Pompe disease (PD). PD is characterized by an abnormal deposit of glycogen in the lysosomes, in which acid alpha-glucosidase (GAA) enzyme is deficient or absent. It is an autosomal recessive disorder and has two forms of presentation: the early infantile-onset Pompe disease (IOPD), and the late-onset Pompe disease (LOPD). The former is distinguished by developing heart disease with high risk of tachyarrhythmia and SCD usually before the first year of life, whereas the later has very rarely been found to present cardiac involvement.

It is a rare disorder with an estimated prevalence in the general population of 1:40,000^76^, with higher prevalence in certain countries such as Taiwan and Austria, in which newborn screening is active^77, 78^.

The severity, tissue manifestations and age of onset, correlate with the nature of the pathogenic variant and degree of residual enzyme activity^79^.

There are three initial pathogenic mechanisms encountered in PD. The most commonly known is the abnormal deposit of glycogen in the lysosomes. The second one is disruption of the normal process of macroautophagy that is needed for the recycling and degradation in the cell, leading to accumulation of toxic materials, as well as oxidative stress. The last one is the resulting damage of the mitochondria, playing an important role in myocardial and skeletal muscle impairment^80^.

In the IOPD form, left ventricular hypertrophy by definition is present before age 12 month and can be as early as in utero. Enzyme replacement therapy (ERT) with alglucosidase alfa treatment should be started as soon as possible, as it has shown to improve cardiomyopathy, ventilation free-survival and overall survival. Without ERT, surveillance is estimated in around two years^81^. Associated with the cardiac condition, life impairment is affected by respiratory and ventilation problems, hypotonia, muscle weakness, feeding difficulties and low growth centiles.

Left ventricular hypertrophy, short PR and LVOT obstruction are the main characteristics. While LVH progresses, the obstruction may worsen and lead to heart failure and respiratory insufficiency, being the most common cause of death^82^.

Among laboratory test performed, serum creatine kinase (CK) as well as urinary oligosaccharides, can raise suspicion about a glycogen storage disorder.

For the diagnosis, determination of GAA deficiency can be made by dry blood spot, but should be confirmed by molecular analysis and genetic testing, in which biallelic pathogenic variants of *GAA* gene must be present.

#### 2.3.2 Danon disease (LAMP2 deficiency)

Danon disease (DD) is a rare HCM phenocopy (1% of HCM^83^), with an X-linked dominant inherit pattern, caused by an inborn disorder in the final step of the autophagic process in the cell, which involves a deficient function of the lysosome-associated membrane protein 2 (LAMP2)^84^.

LAMP2 is located in the lysosome membrane and acts in the process of autophagosome maturation by facilitating the fusion with endosomes. Mutations affecting the *LAMP2* gene that alter the isoform LAMP-2B are responsible for DD. The resulting impaired autophagy will lead to glycogen and non-degraded substance deposit, promoting organ changes and damages. In a similar way to what happened in PD, mitochondrial disturbances play an important role in the pathogenesis of the disease.

##### 2.3.2.1 Diagnostic work-up

2.3.2.1.1 Clinical manifestations

This is a multisystemic disorder, where the extracardiac manifestations of patients with LVH should call clinicians attention to reach the correct diagnosis. Due to the haplo-insufficiency, the characteristic presentation occurs in boys and young males gathering massive increase in cardiac mass, skeletal myopathy and cognitive and psychiatric comorbidities. However, females are also affected but later in age and with different clinical course, as a result of lyonization process. Difference in presentation is synthesized in Table 9. Determining levels of serum creatine kinase or liver function tests can also be of utility.

**Table 9:** Difference in clinical course between sexes. (Note that not many data registries are available and some rates are based on small sample series.)

	Males	Females
Age of onset	13.3 ± 8.0^85^	28.9 ± 4.2^85^
LVH	88–100%^86^	(33%)^86, 87^
DCM	10–12% (late progression)^86, 87^	(28%)^86, 87^
Pre-excitation	69%^86^	27%^86^
Atrial Fibrillation	75–85%^90, 91^	
Heart transplant	20.8 ± 6.7^86^	32.3 ± 14.5^86^
Defibrillator	41%^86^	31%^86^
Death	20.1 ± 5.2^86^	40.2 ± 12.6^86^
Skeletal myopathy	80-90%^86, 87^	33–50%^86, 87^
Gastrointestinal disease	77%^86^	50%^86^
Respiratory disease	50%^86^	16,7%^86^
CK elevation	All^87^	50%^88^
Cognitive impairment	Mild cognitive deficits in most of the patients^89^	
Psychiatric disorder (PD)	69.2% (at least one PD) mood and anxiety the most common^89^	
Retinal problems	69%^86^	64%^86^

2.3.2.1.2 Cardiac assessment

Concentric massive left ventricular hypertrophy in a boy or a young male, with a pre-excitation pattern in the ECG, is the typical presentation of DD. On the contrary, dilated cardiomyopathy is more commonly seen in females. There is a high incidence of atrial fibrillation and ventricular arrhythmias, and the surveillance rate without ICD or heart transplant is really poor in this patients.

Very few reports have been made of MRI characterization of DD. Almost all the cases describe extended LGE, involving different parts of the left ventricle but usually sparing the interventricular septum^92^.

For the definite diagnosis, *LAMP2* gene pathogenic variant is required. Most of the described disease-causing variants are nonsense or frameshift mutations, leading to absence of protein synthesis.

Biopsy of affected tissue, although not obligatory for diagnostic purposes, will show autophagic vacuole accumulation.

##### 2.3.2.2 Treatment.

A close follow up is highly recommended since the diagnosis is done, knowing the poor prognosis and high incidence of life threatening events. Primary prevention of SCD should be assessed based on family history, symptoms, arrhythmias and fibrosis in the MRI, as there are no current specific guidelines in this sense.

Exercise promotion and psychological therapies as well as ophthalmological evaluation ought to be offered to these patients. As in all monogenic disorders, genetic counselling must be offer.

#### 2.3.3 Glycogen storage disease type III (Cori disease, Forbes disease or glycogen debrancher deficiency)

GSD III is a rare autosomal recessive storage disorder, caused by the deficient activity of glycogen debranching enzyme. The LVH is usually found in the IIIa form of GSD type III disease, whereas the IIIb type symptoms are related to the liver. Despite of ventricular hypertrophy, derived cardiac symptoms are not frequently reported. However, there is lack of longitudinal studies evaluating these patients.

Other related features are hepatomegaly, hypoglycemia, hyperlipidemia, variable forms of myopathy and growth retardation^93^.

#### 2.3.4 Glycogen storage disease type IV (Anderson disease, brancher deficiency or amylopectinosis)

It is a rare autosomal recessive metabolic disorder and ventricular hypertrophy as cardiac involvement, caused by a deficiency in glycogen-branching enzyme. There are several forms with different ages of presentation, being the childhood the age of presentation with LVH and myopathy^94^.

## 2.4 PRKAG2 syndrome

### 2.4.1 Introduction

PRKAG2 syndrome (PS) is a rare autosomal dominant inherited disease, with an early onset, caused by a mutation in *PRKAG2*, the gene encoding for the *γ*2 regulatory subunit of the 5′ Adenosine Monophosphate-Activated Protein Kinase (AMPK), which is located in chromosome 7. The pathogenic variants known to cause PS are almost in all cases missense variants.

The main characteristic of PS is its manifestation as a hypertrophic cardiomyopathy phenocopy and a pre-excitation pattern in the surface electrocardiogram. Extracardiac involvement is very rare but severe forms with skeletal myopathy with high levels of creatine kinase (CK) are reported in the literature^95^. Hypertension in the adolescence may be considered an extracardiac expression of the syndrome.

AMPK is regulator of cell metabolism and its activation leads to ATP production by increasing the glucose uptake and fatty acid oxidation and to an inhibition of lipids and proteins synthesis process. In the absence of energy deficit, AMPK should not be activated. In PS, the disorder in the *γ*2 regulatory subunit, which acts as a sensor, entails an aberrant increase of kinase activity.

On the contrary to what initially was thought, only up to 4% of the cardiac mass is due glycogen deposits. In fact, the effect of PRKAG2 in the cardiac growth might be independent from the deposits. A proposed via responsible for myocyte hypertrophy is the increased insulin sensitivity and hyperactivity of Akt, that results in activation of mammalian target of rapamycin (mTOR) and inactivation of forkhead box O transcription factor–signaling pathways^96^. AMPK activation also increases Ca2+ sensitivity of contractile regulation, similarly to what occurs in sarcomeric HCM, sharing common pathophysiological pathways^97^.

#### 2.4.2 Diagnostic work-up

PS is usually diagnosed between the third and fourth decade of life, but severe forms of specific pathogenic variants can manifest as early as the first months of life. There is a high penetrance and one quarter of patients present asymptomatic. There is poor information in natural history of PS reported to date. The few studies available report a high risk of cardiac complications^98^.

**Figure 15.**
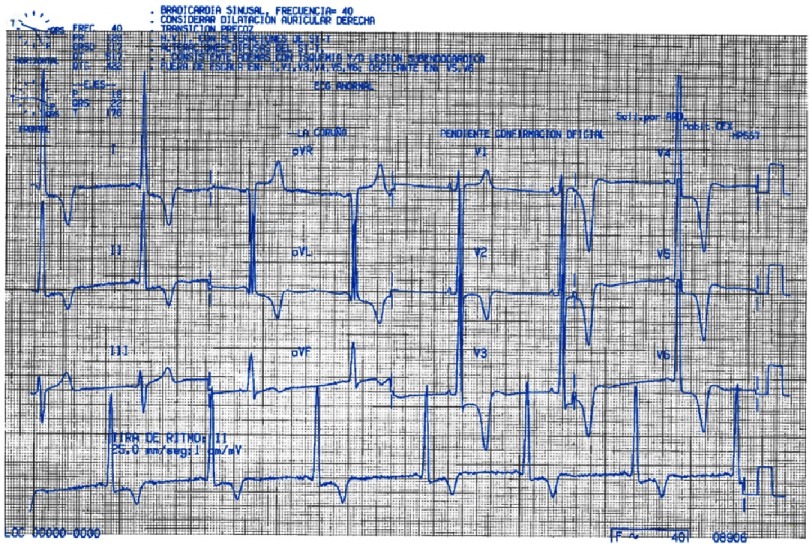
ECG showing pre-excitation pattern and high voltages with T-wave inversion. Concentric left ventricular hypertrophy is present in two thirds of the patients, usually with impaired diastolic function and some with restrictive pattern. Ejection fraction is generally preserved, occasionally with LVOT obstruction. It is also reported the progression to dilated cardiomyopathy. Supraventricular arrhythmias are frequent (38%) mainly atrial flutter and atrial fibrillation. In two thirds of cases there is a form of bradyarrhythmia (AVB or sinus dysfunction) that may need pacemaker implantation. Sudden cardiac death, although rare, can be a form of presentation in a range of 12 to 20% of the patients^98^.

Ventricular pre-excitation is a hallmark that must put the clinician on the track to diagnose PS (Figure 15). A 12% prevalence of patients with storage diseases has been reported among subjects diagnosed with hypertrophic cardiomyopathy and ventricular preexcitation^99^. In PS, the accessory pathways (decremental AV connections or fasciculoventricular pathways) are thought to be a consequence of abnormal atrioventricular septation favoured by the accumulation of glycogen and amylopectin^96, 100^.

The detection of a pathogenic variant in *PRKAG2* gene is the gold standard for the diagnosis of PS^101^. Endomyocardial biopsy can show vacuoles PAS stain (Periodic Acid-Schiff) positive and disarray is an uncommon finding.

The MRI is very useful for early characterization myocardium even in the absence of LVH, when there is no LGE but T1 mapping values may be reduced. In the initial phases of fibrosis development, focal mid inferolateral pattern is the common location, whereas a more diffuse pattern but focused on interventricular septum is present in advance stages. Likewise, as fibrosis increases, T1 mapping values will raise as well^102^.

#### 2.4.3 Management

There are no current specific guidelines for the management of PS, neither for treatment recommendations nor for risk stratification of SCD.

Clinical follow-up is recommended with periodical echocardiogram to look for heart failure (HF) sign and symptoms of disease progression and to establish HF optimal treatment. Early detection of atrial fibrillation or flutter for primary prevention of stroke should be assessed with ECG monitoring, at the time that ventricular arrhythmias or bradyarrhythmias are screened.

It appears to be reasonable to stratify risk of the accessory pathways and undergo ablation on an individualized basis, due to the high incidence of atrial arrhythmias and VF degeneration. Family screening and counselling should always be offered.

There is no treatment currently available for PS, although ongoing research is being developing in the field of genome editing in experimental models with the CRISPR^103^*.* Interestingly, it is also under research the effect of the novel sodium/glucose cotransporter 1 (SGLT) inhibitors used for diabetes mellitus treatment^104^, as AMPK is a modulator for the SGLT activity.

### 2.5 Chloroquine-induced cardiomyopathy

Extended therapies with chloroquine, an antimalarial drug used for lupus treatment, can cause a vacuolar myopathy-like disease, presenting with biventricular hypertrophy and LGE usually in the septum. Mitral, aortic, and tricuspid valve thickening, as well as atrioventricular conduction delay or block, may complete the cardiac phenotype. Retinopathy, myopathy or peripheral neuropathy, can be also part of the syndrome.

These similarities with LSD might be explained by the fact that chloroquine interferes with lysosomal enzyme activity and mitochondrial oxidative metabolism, conducting to glycogen and phospholipids deposit in the same way that occurs in the inborn metabolism disorders reviewed above. Luckily, it might be a reversible condition after drug withdrawal^105^.

## 3 Conclusion

A comprehensive anamnesis is the critical diagnostic tool for facing a patient with left ventricular hypertrophy. Being familiar with the different signs and extracardiac symptoms that characterize the phenocopies is necessary to reach a definite diagnosis (Table 10). The implications of identifying an inborn metabolic disorder or a familiar condition is crucial for the patient’s safety and wellbeing, as well as for family planning^106^.

**Table 10:** Summary of storage disease that can mimic Hypertrophic Cardiomyopathy phenotype.

Cardiomyopathy	Pathogenesis	Substance	Gene & Inheritance Pattern
**Extracellular Deposit**			
Familiar CA	Amyloid fibril formation.	TTR	*TTR* (AD)
Non Familiar CA	Amyloid fibril formation.	TTR, LC, SAA	–
Cardiac Oxalosis	Glyoxylate metabolism	Calcium oxalate	*AGXT,GRHPR, HOGA1* (AR)
**Intracellular Deposit**			
Fabry Disease	Alpha-Galactosidase. Lysosomal storage.	Globotriaosylceramide	*GLA* (X-Link)
Hurler Syndrome	Glycosaminoglycan-degrading enzymes. Lysosomal storage.	Glycosaminoglycans	*IDUA* (AR)
Hunter Syndrome	Glycosaminoglycan-degrading enzymes. Lysosomal storage.	Glycosaminoglycans	*IDS* (X-Link)
Pompe Disease	Defective autophagy lead to Lysosomal storage.	Glycogen	*GAA* (AR)
Cori Disease	Glycogen debrancher deficiency. Lysosomal storage.	Glycogen	*AGL* (AR)
Anderson Disease	Glycogen brancher deficiency. Lysosomal storage.	Amylopectin-like glycogen.	*GBE1* (AR)
Danon Disease	Defective autophagy	Autophagic vacuoles	*LAMP2* (X-Link)
PRKAG2 Syndrome	Dysregulation of AMPK	Glycogen	*PRKAG2* (AD)
Chloroquine-induced myopathy	Drug-induced Lysosomal disorder.	Glycogen	–

**Notes.**

CA Cardiac Amyloidosis TTR Transthyretin LC Light Chains SAA Serum Amyloid A AR Autosomal Dominant AR Autosomal Recessive

Apart from the previously summarized (Table 10), some fatty acid and carnitine disorders can also present with hypertrophy, although these are very rare conditions. Friedreich ataxia, in which abnormal iron regulation and deposition is involved, is explained in other section of this compendium (see *Neuromuscular with HCM*).

Likewise, the differential diagnosis HCM phenocopy should always include other cardiomyopathies such as mitochondrial cytopathies, hypertensive cardiomyopathy, athlete’s heart, infant of diabetic mother’s HCM and rasopathies (Noonan, Costello, Leopard and Cardiofaciocutaneous syndrome).
